# Antiproliferative Modulation and Pro-Apoptotic Effect of BR2 Tumor-Penetrating Peptide Formulation 2-Aminoethyl Dihydrogen Phosphate in Triple-Negative Breast Cancer

**DOI:** 10.3390/cancers15225342

**Published:** 2023-11-09

**Authors:** Laertty Garcia de Sousa Cabral, Cyntia Silva Oliveira, Katielle Albuquerque Freire, Monique Gonçalves Alves, Vani Xavier Oliveira, Jean-Luc Poyet, Durvanei Augusto Maria

**Affiliations:** 1 Laboratory of Development and Innovation, Butantan Institute, Sao Paulo 69310-000, Brazil; laertty.c@usp.br (L.G.d.S.C.); moniquealves@usp.br (M.G.A.); 2 Faculty of Medicine, University of Sao Paulo (FMUSP), Sao Paulo 01246-903, Brazil; 3 Federal University of Sao Paulo (UNIFESP), Sao Paulo 09913-030, Brazil; cyntia.oliveira@unifesp.br (C.S.O.); vani.junior@ufabc.edu.br (V.X.O.); 4 Center for Natural and Human Sciences, Federal University of ABC, Santo Andre 09210-580, Brazil; katiellefreire@gmail.com; 5 INSERM UMRS976, Institut De Recherche Saint-Louis, Hôpital Saint-Louis, 75010 Paris, France; 6 Université Paris Cité, 75006 Paris, France

**Keywords:** nano therapy, triple-negative breast cancer, peptide, monophosphoester

## Abstract

**Simple Summary:**

Triple-negative breast cancer (TNBC) is an aggressive, hard-to-treat form of breast cancer, accounting for about 15 percent of breast cancers. The aim of our study was to assess the potential added value of combining monophosphoester 2-aminoethyl dihydrogen phosphate (2-AEH_2_P), a molecule involved in phospholipid turnover with antiproliferative and pro-apoptotic properties toward cancer cells, and the antitumor BR2 cell-penetrating peptide for TNBC treatment. Using an array of TNBC cells, our data demonstrate that, while possessing limited anti-cancer properties when used as single agents, a drastic synergy was observed with the association 2-AEH_2_P+BR2. Mechanistically, the 2-AEH_2_P+BR2 combination acts by inducing TNBC cells, but not normal cells, apoptosis and by preventing their migration potential through the modulation of cell death-related proteins, reduction of the mitochondrial potential and intracellular redistribution of important components such as the cytoskeleton. Our observations provide a clear rationale for the association of 2-AEH_2_P with the BR2 peptide as a new TNBC treatment.

**Abstract:**

Breast cancer is the most common cancer in women, the so-called “Triple-Negative Breast Cancer” (TNBC) subtype remaining the most challenging to treat, with low tumor-free survival and poor clinical evolution. Therefore, there is a clear medical need for innovative and more efficient treatment options for TNBC. The aim of the present study was to evaluate the potential therapeutic interest of the association of the tumor-penetrating BR2 peptide with monophosphoester 2-aminoethyl dihydrogen phosphate (2-AEH_2_P), a monophosphoester involved in cell membrane turnover, in TNBC. For that purpose, viability, migration, proliferative capacity, and gene expression analysis of proteins involved in the control of proliferation and apoptosis were evaluated upon treatment of an array of TNBC cells with the BR2 peptide and 2-AEH_2_P, either separately or combined. Our data showed that, while possessing limited single-agent activity, the 2-AEH_2_P+BR2 association promoted significant cytotoxicity in TNBC cells but not in normal cells, with reduced proliferative potential and inhibition of cell migration. Mechanically, the 2-AEH_2_P+BR2 combination promoted an increase in cells expressing p53 caspase 3 and caspase 8, a reduction in cells expressing tumor progression and metastasis markers such as VEGF and PCNA, as well as a reduction in mitochondrial electrical potential. Our results indicate that the combination of the BR2 peptide with 2-AEH_2_P+BR2 may represent a promising therapeutic strategy in TNBC with potential use in clinical settings.

## 1. Introduction

Breast cancer accounts for about 25.2% of all female cancers, with an estimated 2 million cases diagnosed worldwide [1]. It presents great heterogeneity, which leads to various clinical outcomes, and is divided into several subtypes, which justifies the need for a better understanding of the disease for the development of more specific and efficacious therapeutic approaches [2]. The triple-negative breast cancer (TNBC) is an aggressive, highly invasive cancer that accounts for about 15% of all breast cancers [3]. Compared to other subtypes, TNBC patients have a shorter tumor-free survival time, with a mortality rate of 40% in the first 5 years after diagnosis and a recurrence rate of 25% after surgery [4,5].

The search for neoadjuvant therapy for TNBC is essential, as it could be used in early-stage localized treatment to promote breast-conserving surgery or for patients for whom surgery is temporarily contraindicated [6,7]. Monophosphoester 2-aminoethyl dihydrogen phosphate (2-AEH_2_P) is a molecule involved in phospholipid turnover, acting as a precursor in the synthesis of membrane phospholipids, including phosphatidylcholine and phosphatidylethanolamine, that participate as ligands or intermediary substrates in lipid signaling pathways [8,9]. 2-AEH_2_P interest as an adjuvant to in vitro and in vivo therapy has already been demonstrated by our group for numerous types of cancer [10,11,12,13,14,15,16]. Indeed, 2-AEH_2_P exerts antiproliferative and pro-apoptotic effects in triple-negative human breast adenocarcinoma cells (MDA-MB-231) [13], human breast cancer cell line with estrogen, progesterone, and glucocorticoid receptors (MCF-7) [11], myelogenous leukemia cell line (K562 e K562 Lucena MDR+) ^13^, murine hepatoma cell line (Hepa-1c1c7) and melanoma-mouse cell line (B16-F10) [14,15,16].

The BR2 peptide is a tumor cell-penetrating derivative of the antitumor peptide buforin IIb, showing higher specificity for tumor cells together with minimized toxicity to normal cells, and that activates endocytic transport pathways distinct from micropinocytosis [17,18]. BR2 peptide translocation involves its binding to membrane gangliosides, which have a more electronegative charge, acting as a primary tumor-specific receptor, followed by proteolytic cleavage, the subsequent release of the RLLR terminal motif, and the binding to a second receptor, neuropilin [19,20]. The transport pathway is activated upon binding to the BR2 RLLR terminal motif to neuropilin-1 or neuropilin-2. [19,20] Such a translocation mechanism can transport varied loads across cell membranes, which is one of the biggest problems in drug administration and targeted therapy.

The antiproliferative and antitumor actions of both 2-AEH_2_P and the BR2 peptide have already been well studied in various cellular cancer models, with very positive results and independently of the resistance profile [10,11,14,15,16,19,21,22]. Interestingly, numerous data indicate that tumor-penetrating peptides have the ability to potentiate the anticancer effect of various drugs [17,18,23,24], and the association of the BR2 peptide with chemotherapeutic agents or toxins has demonstrated strong synergic effects in various cellular settings [20,25,26]. These observations provide a rationale for evaluating the interest of combining the tumor-specific peptide BR2 with 2-AEH_2_P as a new anti-cancer strategy.

In this study, we assessed the therapeutic potential of the 2-AEH_2_P+BR2 association, its antiproliferative and antitumor action, as well as the possible mechanism of action of the association of death in the context of TNBC.

## 2. Materials and Methods

### 2.1. Cell Culture

Human breast adenocarcinoma cell lines MDA-MB-231 (ATCC CRM-HTB-26), murine breast adenocarcinoma 4T1 (ATCC CRL-2539), human umbilical vein endothelial cells HUVEC (ATCC CRL-1730), and normal human fibroblast FN1. The cells were cultivated in RPMI-1640 medium (2 mg/mL glucose; LGC Biotecnologia, Cotia, SP, Brazil). The medium was supplemented with sodium bicarbonate (24 mM), 0.01% antibiotics, and 10% fetal bovine serum (FBS) (Cultilab, Campinas, SP, Brazil). They were grown in an atmosphere of 5% CO_2_ at 37 °C. Cell viability was evaluated using the Trypan Blue exclusion test, and values greater than 94% were considered.

### 2.2. Cytotoxicity Assessment by the MTT Colorimetric Assay

Cells in 96-well plates (10^5^ cells/mL) were treated with different concentrations of the BR2 peptide, 2-AEH_2_P, and the combination of 2-AEH_2_P+BR2 for 24 h or 48 h. The supernatant was then removed, 100 µL of 5 mg/mL MTT (Calbiochem—Darmstadt, Germany) in RPMI-1640 medium was added, and the cells were incubated for 3 h at 37 °C in an atmosphere containing 5% CO_2_. Subsequently, the supernatant was removed, and 100 µL of methanol was added to dissolve the formazan crystals. The absorbance quantification was performed in an ELISA reader at 540 nm. The IC50 was determined as the concentration that induces toxicity in 50% of the cells after 24 h and 48 h at different treatments to assess the effect of time/concentration. For the association 2-AEH_2_P+BR2, half of the IC50 values of 2-AEH_2_P (fixed concentrations) obtained from each cell, with increasing concentrations of BR2 peptide, were used to assess whether the peptide concentrations reduced and potentiated the cytotoxic effect.

### 2.3. Cell Viability Analysis Using Trypan Blue Exclusion

Tumor or normal cells were incubated in 24-well plates at 10^4^ cells/mL for 24 h, 48 h, 72 h, and 96 h and treated with BR2 peptide, 2-AEH_2_P, and the association of 2-AEH_2_P+BR2 at IC50 values of the respective tumor cells. Control cells (HUVEC and FN1) were treated at the IC50 values of the tumor cells. After the treatment, the supernatant was discarded, and 200 µL of trypsin was added, followed by an incubation of 5 min in an incubator with an atmosphere containing 5% CO_2_ at 37 °C. Subsequently, the content was neutralized with 400 µL of RPMI-1640 culture medium (LGC Biotecnologia, Cotia, SP, Brazil) containing 10% FBS. The content was transferred to 15 mL tubes and centrifuged at 1200 rpm for 5 min. The supernatant was removed, and 1 mL of RPMI-1640 culture medium was added to resuspend the pellet. The viability was assessed using Trypan Blue staining.

### 2.4. Wound-Healing Assay

Cells were plated at 10^5^ per well in 24-well culture plates until reaching 90% confluence. With the aid of a 1000 μL pipette tip, a vertical or horizontal wound was created. The assays were performed in the presence of actinomycin C (2 ng/mL) to prevent cellular proliferation. A Nikon Eclipse TE300 inverted microscope equipped with an environmental camera (In Vivo Scientific, LLC, Salem, SC, USA) was used to capture images with a Nikon Plan Apo 4×/0.2 objective. The location of the photographed cells was recorded and saved with Velocity software 3.8 (Velocity Software, Inc., Mountain View, CA, USA), allowing the same location to be photographed at 0 and 24 h. The effectiveness of inhibition was estimated by the relative distance of wound closure.

### 2.5. CFSE-DA Proliferation Assay

Human MDA MB-231 and murine 4T1 triple-negative breast cancer tumor cells were incubated in 24-well plates, 10^5^ cells/mL for 24 h. The treated and control groups were incubated with the carboxyfluorescein marker (CFSE-DA Thermo Fisher, KITC34571, Waltham, MA, USA). CFSE-DA was diluted in PBS containing 0.1% human albumin and added to the cells. After 24 h, the cells were trypsinized, transferred to a conical tube, and centrifuged at 1500× *g* rpm for 5 min. The supernatant was then discarded, and the pellet resuspended in 1 mL of 4% paraformaldehyde in PBS for 30 min. The cells were then centrifuged, the supernatant discarded, and the cell pellet resuspended in 200 μL of FACs buffer. The reading was performed in a FACScanto flow cytometer (BD) with the number of events (10,000 events), and the histograms were acquired and analyzed by ModFit LT 5.0 software.

### 2.6. Evaluation of the Number of Cells Expressing Markers Involved in Apoptosis and Progression by Flow Cytometry

Following the indicated treatments, 100 μL aliquots of the MDA-MB 231 tumor cells at a concentration of 10^6^ cells/mL were incubated with Triton X-100 (0.1% final) for 30 min. The mixture was then centrifuged at 1500 rpm for 5 min, the pellet resuspended in 200 μL of FAC’s buffer and incubated with 1 μg of specific antibody to either caspase 3, caspase 8 and P53 conjugated with phycoerythrin-PE for 1 h at 4 °C. Antibodies to markers involved in metastatic regulators such as VEGF and EGF conjugated to phycoerythrin-PE were also used. The cells were then centrifuged at 1500× *g* rpm, washed with PBS, and the pellet was resuspended in 200 µL of FACS buffer containing 0.1% paraformaldehyde. The analysis of marker expression was performed in a FACScanto flow cytometer–BD (Franklin Lakes, NJ, USA) at FLH-1 fluorescence intensity (10,000 events), and DotPlots were acquired and analyzed by Cell-Quest-DB 2.0 software.

### 2.7. Analysis of Mitochondrial Electrical Potential by Flow Cytometry

Cells (10^6^ cells/mL) were grown in 24-well plates for 24 h. Treatments were performed for a period of 24 h. Cells were then washed in fresh RPMI-1640 medium, trypsinized, and resuspended in 200 nM MitoRed (Sigma-Aldrich, Waltham, MA, USA) solution in complete RPMI-1640 medium and incubated for 1 h in the dark at 37 °C. Subsequently, the cells were fixed in 1 mL of 4% paraformaldehyde for 30 min. The cells were then centrifuged, and the pellet was resuspended in 200 μL of FACS Flow buffer. The reading and analysis of Rhodamine-123 staining on cells were performed in a FACScanto flow cytometer (BD) at FL1-H fluorescence intensity (10,000 events), and the histograms were acquired and analyzed by the Cell-Quest-BD 2.0 software.

### 2.8. Statistical Analysis

The data obtained were expressed as mean ± standard deviation. The tabulated and analyzed results were processed using the GraphPad software, Version 5.0 and Version 7.0. The analysis was performed by comparing three or more groups with non-parametric distribution by means of the analysis of variance (ANOVA), followed by the Tukey-Kramer test of multiple comparisons, considering *p* ≤ 0.05 as the critical level of significance.

## 3. Results

### 3.1. Determination of Cytotoxic Activity

**Figure 1:**
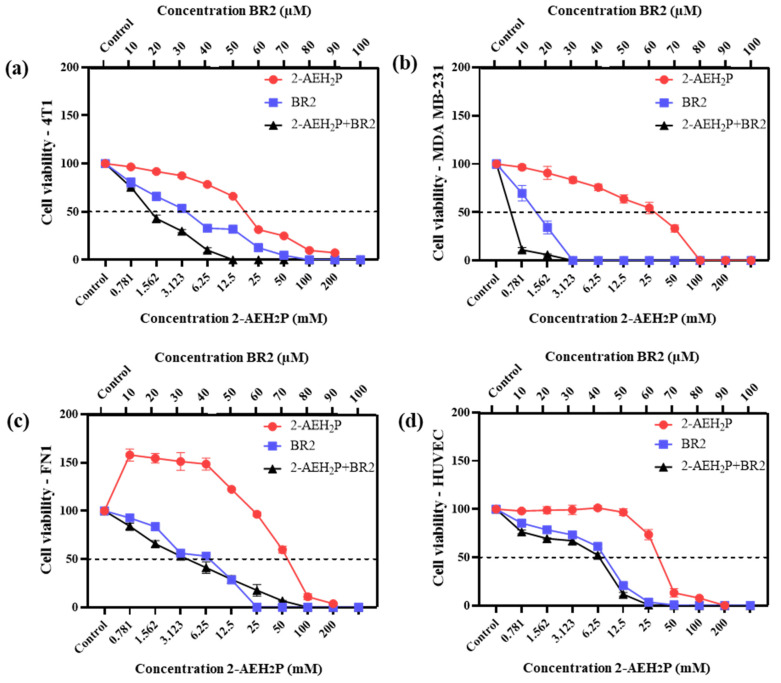

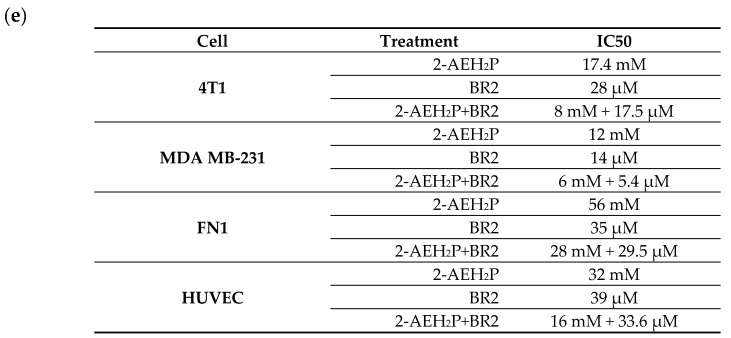
Cytotoxicity assessment of 2-AEH_2_P, BR2 peptide, or the 2-AEH_2_P-BR2 peptide treatments upon 24 h exposure. (**a**) Line graph showing the cytotoxic effect of treatments on 4T1 tumor cell; (**b**) Line graph showing the cytotoxic effect of treatments on MDA-MB-231 tumor cell; (**c**) Line graph showing the cytotoxic effect of treatments on FN1; (**d**) Line graph showing the cytotoxic effect of treatments on HUVEC. Line graphs showing the mean ± SD correlation of four independent experiments. (**e**) Summary table based on the IC50 values determined for the different treatments from the proliferation assays on tumor and normal cells.

**Figure 2:**
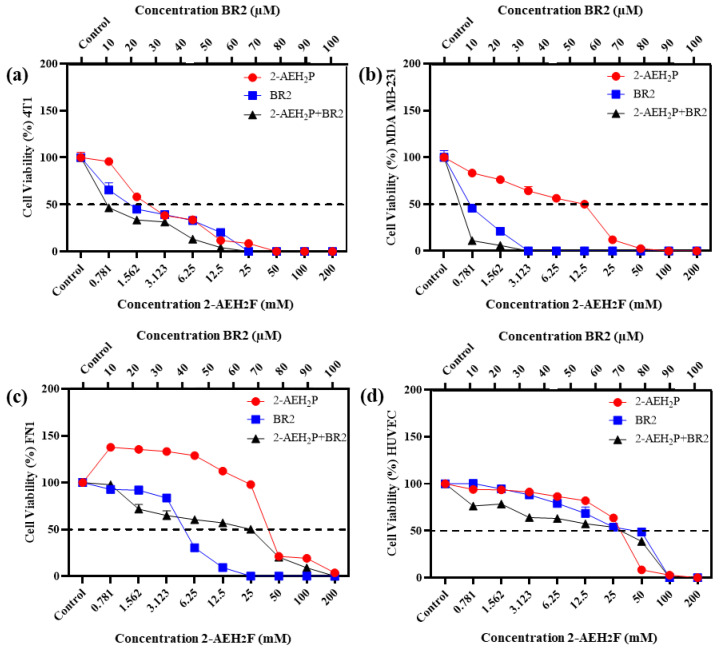

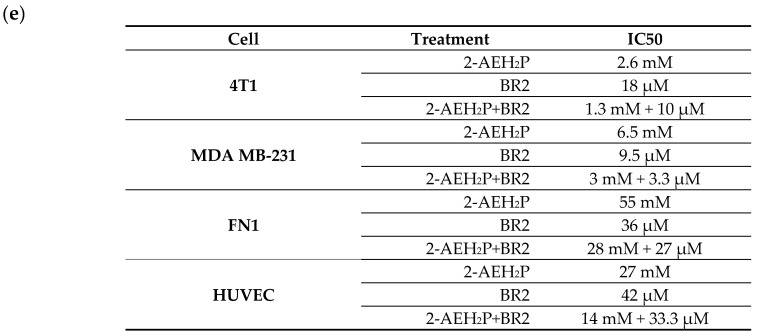
Cytotoxicity assessment of 2-AEH_2_P, BR2 peptide, or the 2-AEH_2_P-BR2 peptide treatments upon 48 h exposure. (**a**) Line graph showing the cytotoxic effect of treatments on 4T1 tumor cell; (**b**) Line graph showing the cytotoxic effect of treatments on MDA-MB-231 tumor cell; (**c**) Line graph showing the cytotoxic effect of treatments on FN1; (**d**) Line graph showing the cytotoxic effect of treatments on HUVEC. Line graphs showing the mean ± SD correlation of four independent experiments. (**e**) Summary table based on the IC50 values determined for the different treatments from the proliferation assays on tumor and normal cells.

Upon exposure to either the peptide BR2, 2-AEH_2_P or the association 2-AEH_2_P+BR2, 4T1, and MDA-MB-231 TNBC cells showed reduced adhesion and morphological changes such as cytoplasmic retraction, an increase in cytoplasmic volume and cell fragmentation. The IC50 values for 2-AEH_2_P in murine 4T1 tumor cells were 17.4 mM and 2.6 mM for 24 h and 48 h treatments, respectively. The peptide BR2 and the association 2-AEH_2_P+BR2 significantly promoted cytotoxicity at all concentrations tested when compared to 2-AEH_2_P, with IC50 of 28 µM and 18 µM for 24 h and 48 h treatments, respectively, for the BR2 peptide and 17.5 µM and 10 µM for the association 2-AEH_2_P+BR2 (Figure 1a and Figure 2a). The IC50 values for human MDA-MB-231 tumor cells for 24 h and 48 h treatments were 12 mM and 6.5 mM for 2-AEH_2_P, 14 µM and 9.5 µM for the BR2 peptide, and 5.4 µM and 3.3 µM for the 2-AEH_2_P+BR2 association, respectively, indicating higher sensitivity compared to the 4T1 cells (Figure 1b and Figure 2b).

Normal human endothelial cells (HUVEC) and normal human fibroblast (FN1) exhibited substantially lower sensitivity to all treatments compared to MDA-MB-231 and 4T1 tumor cells. However, there were some changes in their morphology depending on the treatment. 2-AEH_2_P did not induce significant cytotoxicity in HUVEC cells, with an IC50 of 32 mM and 27 mM for 24 h and 48 h treatments, respectively (Figure 1d and Figure 2d). Treatment with the BR2 peptide resulted in significant cytotoxicity only at the highest concentrations tested, with an IC50 of 39 µM and 42 µM upon 24 h and 48 h treatment, respectively. Of note, after 48 h of treatment, the cells proliferated again, resulting in an IC50 value higher than for the 24 h treatment period. The association of 2-AEH_2_P+BR2 resulted in an IC50 of 33.6 µM and 33.3 µM for the BR2 peptide for 24 h and 48 h treatments, respectively, with 2-AEH_2_P fixed at a concentration of 16 mM (Figure 1c and Figure 2c). In FN1 cells, 2-AEH_2_P did not induce significant cytotoxicity, while at concentrations of 0.7 mM to 12.5 mM, it induced cell proliferation (Figure 1c and Figure 2c). The BR2 peptide exhibited IC50 of 35 µM and 36 µM for 24 h and 48 h treatments, respectively, comparable to that observed for HUVEC. The association 2-AEH_2_P+BR2 exhibited an IC50 of 29.5 µM and 27 µM for the BR2 peptide for 24 h and 48 h treatments, respectively, with 2-AEH_2_P fixed at a concentration of 28 mM (Figure 1d and Figure 2d,e).

Our data indicate that TNBC cells are substantially more sensitive to 2-AEH_2_P and the BR2 peptide than normal cells and that the combination of the compounds increases their cytotoxic effect.

### 3.2. Inhibitory Effect of the Growth Curve on Tumor Cells

**Figure 3:**
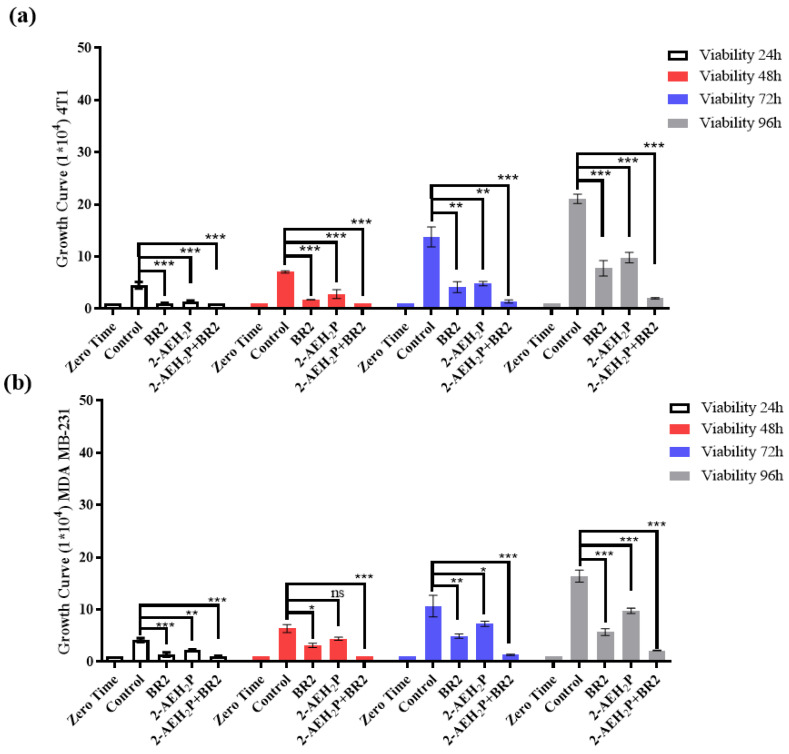

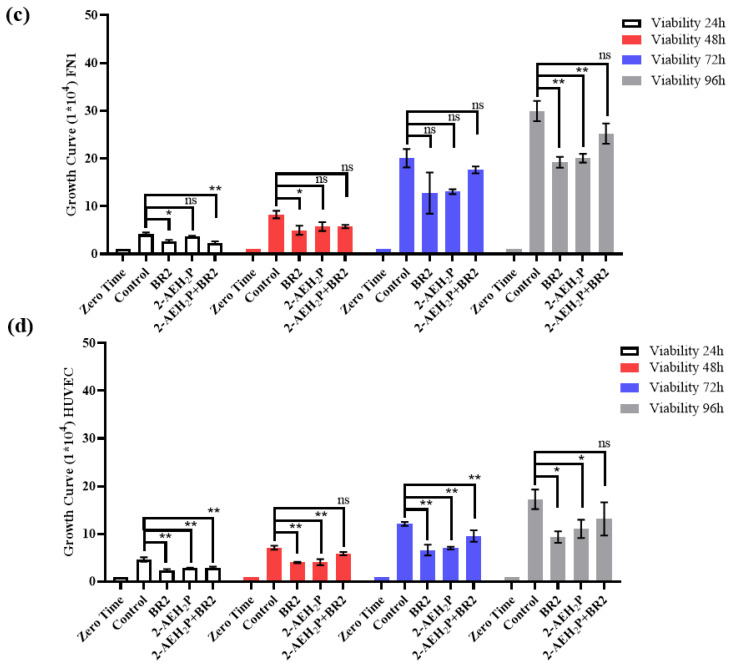
Growth curve of tumor and normal cells as assessed by trypan blue staining (**a**) Bar graphs show the proliferative capacity of 4T1 tumor cells; (**b**) Bar graphs show the proliferative capacity of MDA MB-231 tumor cells; (**c**) Bar graphs show the proliferative capacity of FN1 fibroblast normal cells; (**d**) Bar graphs show the proliferative capacity of HUVEC normal cells. Bar graphs showing the mean ± SD correlation of three independent experiments. Statistical differences were obtained by ANOVA and Tukey-Kramer multiple comparison test. * *p* < 0.05, ** *p* < 0.01 and *** *p* < 0.001. ns = not significant.

We next evaluated the proliferative capacity of human breast adenocarcinoma cell lines MDA-MB-231 and murine breast adenocarcinoma cell lines 4T1 in the presence of 2-AEH_2_P and BR2 peptide, either separated or combined, for up to 96 h of exposure at the IC50 concentrations previously determined. Both adhesion and proliferative capacities of the 4T1 tumor cells were reduced upon all treatments, except for the treatment with 2-AEH_2_P for 24 h. However, a sharp reduction in proliferative capacity was observed for a 48 h treatment. A 72 h treatment resulted in a proliferation reduction of 41.21 ± 3.6% for 2-AEH_2_P, 66 ± 3.1% for the BR2 peptide, and 76 ± 5.8% for the association 2-AEH_2_P+BR2 (Figure 3a). Upon 96 h of exposure, 4T1 tumor cells’ proliferative capacity was slightly increased for both treatments, especially for the 2-AEH_2_P treatment (Figure 3a). MDA-MB-231 tumor cells were less sensitive to 2-AEH_2_P treatment up to 72 h but exhibited a proliferative reduction of 47.3 ± 2.7% upon a 96 h treatment. The BR2 peptide and the 2-AEH_2_P+BR2 association were more effective up to 72 h post-treatment, with a proliferative reduction of 63.6 ± 3.1% for the BR2 peptide and 76.5 ± 4.1% for the 2-AEH_2_P+BR2 association, but, as observed with the 4T1 tumor cells, MDA-MB-231 tumor cells proliferation increased after 96 h of exposure with percentage values of 39.4 ± 3.5% for the peptide BR2 and 51.5 ± 2.9% for the association 2-AEH_2_P+BR2. This increase in cell proliferation might be explained by the proteolytic degradation of the peptide by serum proteases and peptidases after a 96 h incubation (Figure 3b).

We then evaluated the impact of 2-AEH_2_P, BR2 peptide, and 2-AEH_2_P+BR2 association treatment on normal human endothelium and normal human fibroblast proliferation. The compounds were used at concentrations corresponding to the IC50 of 4T1 and MDA MB-231 tumor cells. When FN1 cells were treated with compound concentrations corresponding to the IC50 of 4T1 tumor cells, a reduction in proliferative capacity was observed in the first three treatment periods (24 h, 48 h, and 72 h) upon treatment with BR2 peptide alone and for the 2-AEH_2_P+BR2 association, with a reduction of 23.7 ± 2.8% and 44.5 ± 4.7%, respectively (Figure 3c). A proliferation reduction was only observed for 2-AEH_2_P in the 24 h and 48 h treatment periods, with a reduction of 49 ± 1.9% upon 48 h of exposure (Figure 3a). No significant proliferative change was observed for either treatment after 96 h of exposure. A reduction in the proliferative potential for the HUVEC cell line was observed within 24 h when treated with BR2 peptide and 2-AEH_2_P+BR2 association (Figure 3d). A reduction of 25 ± 0.9% for the BR2 peptide and 34.3 ± 3.4% for the 2-AEH_2_P association was observed following a 72 h treatment, with no change being observed for the 96 h treatment (Figure 3d). Of note, no reduction in the proliferative potential was observed for both FN1 and HUVEC cells when treated at concentrations corresponding to the IC50 of MDA-MB-231 tumor cells at either treatment period (Figure 3c,d).

### 3.3. Inhibition of Breast Cancer Migration by Wound-Healing Assay

**Figure 4:**
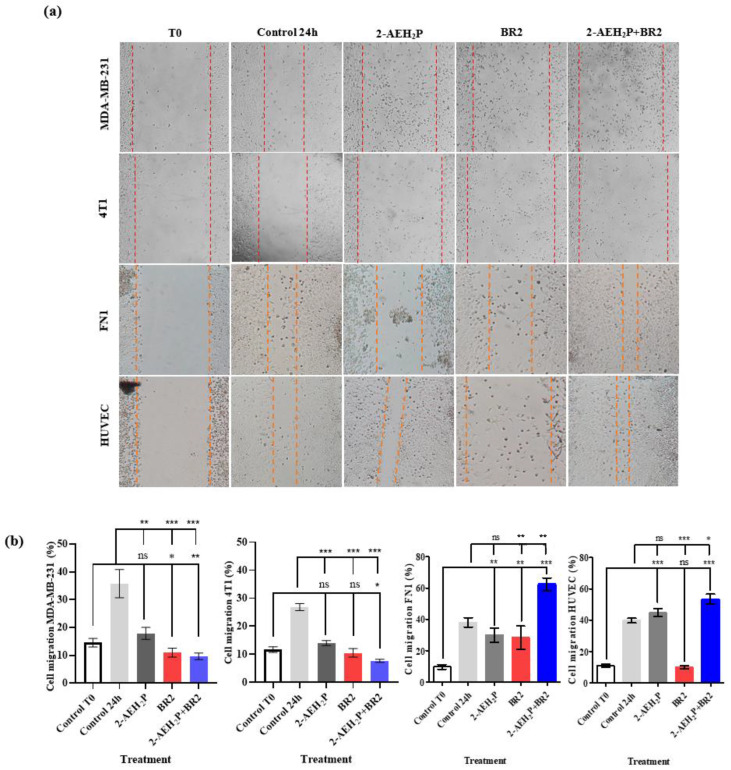
Evaluation of inhibition of migration potential of MDA-MB-231 and 4T1 breast adenocarcinoma cell lines by wound healing assay. (**a**) Photomicrographs of MDA-MB-231 and 4T1 TNBC cells and FN1 and HUVEC normal cells. Vertical lines indicate the wound formed and evolution for each treatment period (**b**) Bar graphs showing the average correlation ±SD of three independent experiments on the ability to inhibit migration of tumor cells exposed to the indicated treatments. Statistical differences were obtained by ANOVA and Tukey-Kramer multiple comparison test. * *p* < 0.05, ** *p* < 0.01 and *** *p* < 0.001. ns = not significant.

We then investigated the impact of 2-AEH_2_P, BR2 peptide, and association 2-AEH_2_P+BR2 on the migration capacity of the triple-negative human MDA-MB-231 and murine 4T1 tumor cells by wound healing assay. A sharp reduction in migration potential was observed for MDA-MB-231 tumor cells for all treatments, with percentage reductions of 50 ± 5.8% for the treatment with 2-AEH_2_P, 69.2 ± 4.3% for the BR2 peptide, and 73.1 ± 2.8% for the 2-AEH_2_P+BR2 association (Figure 4a,b). Similar results were observed for 4T1 tumor cells, with a reduction in the migration capacity of 47.7 ± 2.2% for 2-AEH_2_P, 60.8 ± 3.6% for the BR2 peptide and 71.2 ± 1.8% for the 2-AEH_2_P+BR2 association (Figure 4a,b). Importantly, treatment of normal FN1 cells resulted in a modest to statistically non-significant reduction of cellular migration when 2-AEH_2_P or the BR2 peptide were used separately, whereas, surprisingly, an increase in FN1 cells migration was observed when the compounds were combined (Figure 4a,b). No significant changes in cellular migration were observed when HUVEC cells were treated with 2-AEH_2_P or 2-AEH_2_P +BR2, whereas, surprisingly, a substantial decrease of cell motility was evidenced when the BR2 peptide was used separately, indicating that the presence of 2-AEH_2_P might “counterbalance” the negative impact BR2.

These results demonstrate a strong anti-migration effect for the 2-AEH_2_P+BR2 peptide association for TNBC cells, but not normal cells, that is superior to that of the isolated compounds.

**Figure 5:**
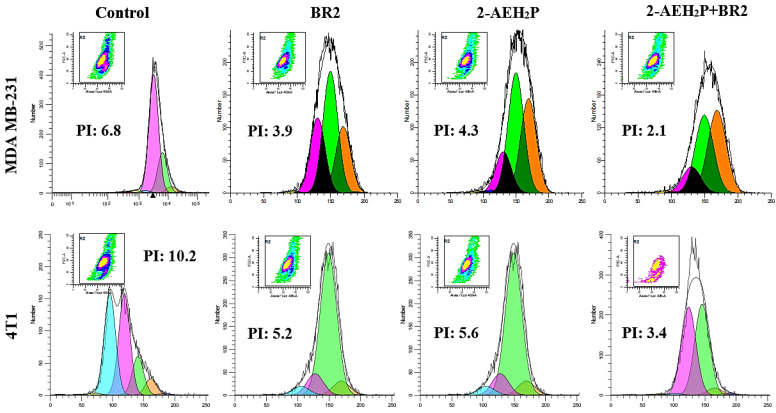
Evaluation of the proliferative index of human MDA MB-231 and murine 4T1 triple-negative breast cancer tumor cells by flow cytometry. The cells were treated with the indicated compounds for a period of 24 h. Representative histograms of the proliferative index were obtained using the WinMDI 5.0 Software. Results expressed as mean ± SD of three experiments independent of the proliferative index of tumor cells.

We next evaluated the proliferative index (PI) of human MDA MB-231 and murine 4T1 triple-negative breast cancer cells when treated with 2-AEH_2_P, BR2 peptide and their association by flow cytometry. A strong reduction in the proliferative index was observed upon treatment of the MDA MB-231 tumor cells, with proliferative index reductions of 42.6 + 2.1%, 36.7 + 1.7%, and 69.1% upon treatments with the BR2 peptide, the 2-AEH_2_P and the 2-AEH_2_P+BR2 association respectively (Figure 5). The proliferative index reductions for the 4T1 tumor cell upon treatment with the BR2 peptide were 49 + 1.1%, 45 + 2.5% for 2-AEH_2_P and 66.6 + 2.7% for the 2-AEH_2_P+BR2 association (Figure 5). Therefore, our data clearly demonstrate a superior effect on TNBC cell proliferation for the 2-AEH_2_P+BR2 association compared to the effect observed when the compounds are used separately.

### 3.4. Modulation of the Mitochondrial Electrical Potential (ΔΨm) of Tumor Cells

**Figure 6:**
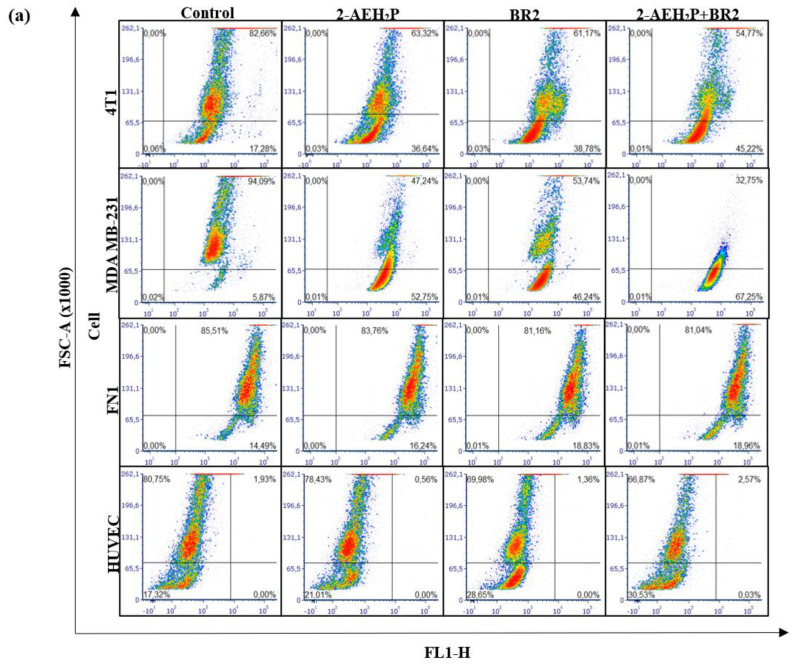

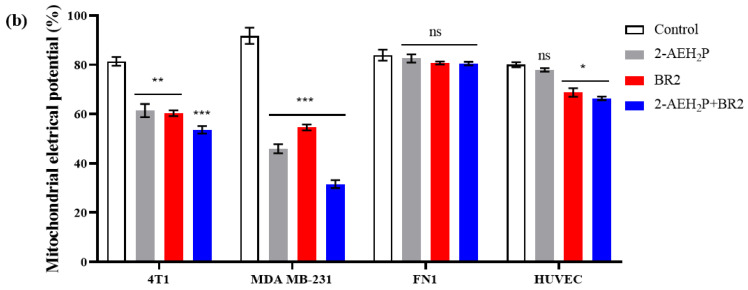
Analysis of mitochondrial electrical potential (ΔΨm) in MDA-MB-231 and 4T1 breast adenocarcinoma cell lines and FN1 normal human fibroblast and HUVEC endothelial cells. (**a**) Density Plots of tumor and normal cells with mitochondria stained with MitoRED and analyzed by flow cytometry; (**b**) Bar graph showing ΔΨm expressed as mean ± SD of three independent experiments. Statistical differences were obtained by ANOVA and Tukey-Kramer multiple comparison tests. * *p* < 0.05, ** *p* < 0.01 and *** *p* < 0.001. ns = not significant.

We then sought to determine whether the BR2 peptide, 2-AEH_2_P, and the 2-AEH_2_P+BR2 association could also influence the metabolic activity of TNBC cells. Analysis of mitochondrial electrical potential (ΔΨm) by the fluorescence intensity of the photomicrographs obtained by confocal microscopy showed a reduction in the ΔΨm of 4T1 tumor cells for all treatments, the reduction being more significant for the 2-AEH_2_P+BR2 association with reduction of 27.75 ± 2.9%, whereas treatment with 2-AEH_2_P and the BR2 peptide resulted in a reduction of ΔΨm of 21.18 ± 2.1% and 21 ± 4%, respectively (Figure 6a,b). The MDA-MB-231 tumor cell exhibited a greater impact on the mitochondrial electrical potential upon treatments, with observed reduction values of 45.8 ± 3.1% for the treatment with 2-AEH_2_P, 37.1 ± 2.3% for the BR2 peptide, and 60.1 ± 2.1% for the association 2-AEH_2_P+BR2 (Figure 6a,b). These results clearly demonstrate that the association of AEH_2_P+BR2 peptide strongly enhances the influence of the compounds on TNBC cells’ mitochondrial membrane potential loss.

Interestingly, normal human fibroblast and normal human endothelial cells exhibited a lesser reduction in ΔΨm upon treatments. There was no reduction in ΔΨm for normal human fibroblast FN1 cells in any treatment (Figure 6a,b). HUVEC cells showed a reduction in the ΔΨm for treatments with peptide BR2, and the effect of the association 2-AEH_2_P+BR2 was substantially lower than that observed in cancer cells, with percentage values of 11.2 ± 3.3% and 13.6 ± 1.3%, respectively (Figure 4a,b). These results clearly demonstrate that the association of AEH_2_P+BR2 peptide strongly enhances the influence of the compounds on TNBC cells’ mitochondrial membrane potential loss.

### 3.5. Analysis of Markers Involved in Cell Death, Angiogenesis, and Proliferation Pathways in MDA MB-231 Tumor Cells upon 2-AEH_2_P, BR2 Peptide or 2-AEH_2_P+BR2 Treatments

**Figure 7:**
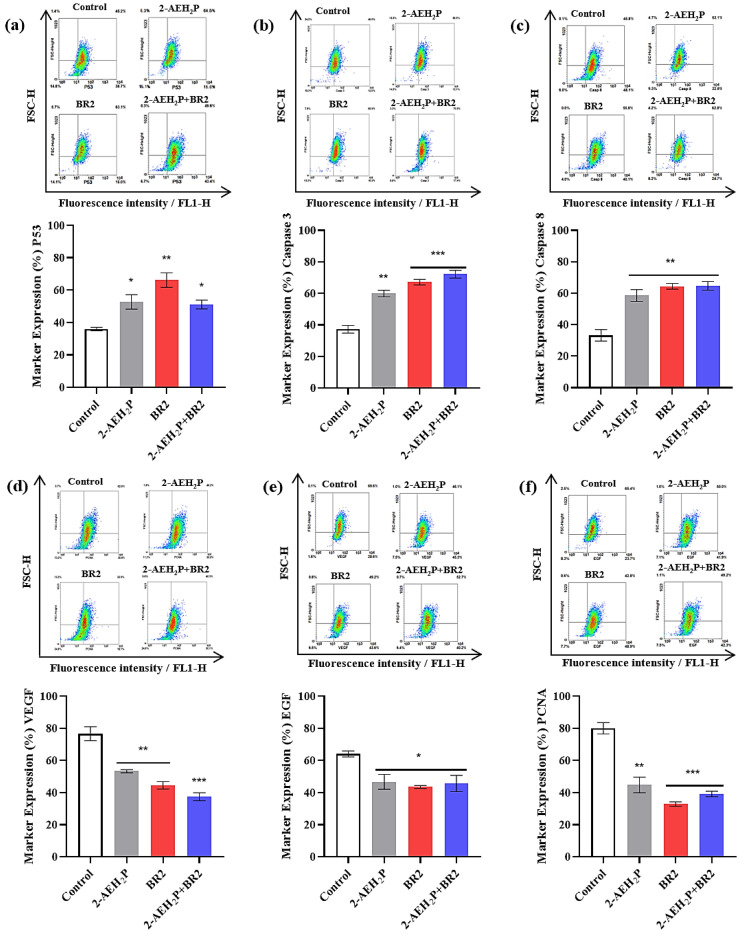
Analysis of the expression of markers involved in cell death, angiogenesis, and proliferation in breast adenocarcinoma cell lines MDA-MB-231 upon treatment with 2-AEH_2_P, BR2 peptide, or 2-AEH_2_P+BR2. (**a**) Analysis of P53 expression; (**b**) Analysis of caspase 3 expression; (**c**) Analysis of caspase 8 expression; (**d**) Analysis of VEGF receptor expression; (**e**) Analysis of EGF expression; (**f**) Analysis of PCNA expression. Bar graphs showing protein expression level as mean ± SD from three independent experiments. Representative density plots show the distribution of cell numbers with fluorescence intensity. Statistical differences were obtained by ANOVA and Tukey-Kramer multiple comparison tests. * *p* < 0.05, ** *p* < 0.01 and *** *p* < 0.001.

We then developed a flow cytometry approach to investigate whether 2-AEH_2_P or BR2 peptide, used alone or combined, might affect TNBC cells’ expression of cellular markers involved in cell death, angiogenesis, and tumor proliferation. There was a considerable increase in cells expressing P53, especially for the treatment with the BR2 peptide, with an increase of 45.7 ± 5.8%, whereas treatments with 2-AEH_2_P or the association 2-AEH_2_P+BR2 resulted in P53 expression increases of 31.8 ± 5.6% and 29.7 ± 3.4%, respectively (Figure 7a). Interestingly, a significant increase in cells expressing caspase 3 and 8 was observed following all treatments, the treatment with the BR2 peptide and association being more effective at increasing caspase 3 expression. Indeed, as shown in Figure 7b, the BR2 peptide and the 2-AEH_2_P+BR2 association treatments increased expression of caspase 3 by 44.5 ± 2.8% and 48.4 ± 3.1%, respectively, whereas a 37.8 ± 3.5% increase for caspase 3 expression was observed upon 2-AEH_2_P treatment. All treatments promoted similar increases in caspase 8 expression, with values of 42.9 ± 4.7% for 2-AEH_2_P, 48.4 ± 2.3% for the BR2 peptide, and 48.6 ± 3.5% for the 2-AEH_2_P+BR2 association (Figure 7c).

Interestingly, there was a considerable reduction in cells expressing total VEGF receptor after all treatments, the 2-AEH_2_P+BR2 association being the most effective, reducing VEGF levels by 51 ± 3.1%, whereas 2-AEH_2_P and the BR2 peptide reduced VEGF levels by 30.2 ± 4.8% and 42.2 ± 3%, respectively (Figure 7d). A reduction in EGF expression was also observed for all treatments, with a reduction of 27.1 ± 5.9%, 32.1 ± 1.2% for 2-AEH_2_P the BR2 peptide, respectively, and 28.8 ± 6.2% for the association 2-AEH_2_P+BR2 (Figure 7e). Finally, a sharp reduction of cell expression proliferating cell nuclear antigen (PCNA) was observed for treatments with peptide BR2 and 2-AEH_2_P+BR2 association, with values of 59 ± 1.9% and 51.1 ± 2.2%, respectively, whereas a 44 ± 6% reduction on PCNA expression was noted for 2-AEH_2_P treatment (Figure 7f).

## 4. Discussion

TNBC is a subtype associated with a more aggressive clinical outcome compared to other breast cancer subtypes, being associated with a higher rate of progression, lower tumor-free survival, and higher mortality, therefore representing an ongoing challenge [27]. In the present study, we evaluated the pro-apoptotic, antiproliferative, and migration inhibitory capacity of 2-AEH_2_P, BR2 peptide, and the association 2-AEH_2_P+BR2 in MDA-MB-231 and 4T1 breast adenocarcinoma cell lines. We show that whereas both 2-AEH_2_P and the BR2 peptide induced a significant inhibition of breast cancer cell migration and proliferation, a clear pharmacological synergic effect was observed with the association of 2-AEH_2_P+BR2. The treatments induced important changes, modulating proteins involved in cell death, reduction of mitochondrial electrical potential, and intracellular redistribution of important components such as the cytoskeleton. The ability of the AEH_2_P+BR2 association to induce apoptosis was evidenced by an observed upregulation of caspase 3 and caspase 8, with again a marked increase compared to the compounds used separately.

Previous studies have demonstrated that the BR2 peptide possesses antitumor toxicity toward a number of cell lines, such as HepG2 (hepatocellular carcinoma) [26], HeLa (human cervical cancer), and B16-F10 (murine melanoma) [28]; HCT116 (human colon cancer) [20]; MCF-7 (human breast cancer) [29]. Importantly, the BR2 peptide exhibits 4- to 5-fold lower toxicity toward normal cells compared to cancer cells, not causing major damage when administered at tumor cells-efficient concentrations, thus providing a large therapeutic window. This characteristic is due to the ability of the peptide to exert its antitumor activity by preferentially binding to and penetrating the cancer cell’s membrane, accumulating in the cytoplasm and the nucleus within 30 min after exposure, where it interacts with macromolecules such as nucleic acids and proteins, and finally, inhibiting cellular mechanisms vital for progression and maintenance of cancer cells [30,31]. Furthermore, the BR2 peptide can induce the intrinsic pathway of apoptosis by activating caspase 3 and caspase 9 in cancer cells [32]. Finally, the BR2 peptide has been demonstrated to be effective in modulating innate immunity while exhibiting a high capacity to promote cell cycle arrest in tumor cells [20].

Our previous data suggest that the sensitivity of tumor cells to monophosphoesters is independent of their aggressive molecular profile or multidrug resistance [11,12,33,34,35]. Among those compounds, the monophosphoester 2-aminoethyl dihydrogen phosphate has demonstrated broad antitumor potential, inducing cytotoxicity in several tumor cell lines, such as EAT (Ehrlich ascites tumor) [12], B16F10 cells (murine melanoma) [34], MCF-7 cells (human breast adenocarcinoma) [13], MDA-MB-231 cells (human triple-negative breast cancer) [14], Skmel-28 and Mewo (human melanoma) [11,15]; Hepa1c1c7 cells (hepatocarcinoma) [16,36], K562 and K562-Lucena (Chronic myeloid leukemia) cells [10,25,35]. Although monophosphoesters are a class of molecules with great antitumor potential, some side effects such as gastrointestinal toxicity, high hemolytic percentage, and hepatic and renal dysfunction have been described for several commercial monophosphoesters of this family (edelfosine, miltefosine, and ilmofosine) [29], thus hampering their use in the clinic.

Our work demonstrates that the association of 2-AEH_2_P+BR2 showed promising results at low concentrations for both compounds, exhibiting high cytotoxicity toward tumor cells but not normal cells. Recently, several studies have demonstrated the effectiveness of the BR2 peptide in the targeted delivery of other molecules to tumor cells. For instance, whereas diphtheria toxin catalytic and translocation domains (DT_386_) only possess low toxicity toward cancer cells, a fusion protein in which DT_386_ is linked to the BR2 peptide exhibits strong cytotoxic effects toward human breast cancer (MCF-7) and cervical cancer (HeLa) cells, with no significant cytotoxic effects on normal cells [37]. In line with these results, association of IL 24 with the BR2 peptide strongly increased IL 24-related cytotoxicity toward cancer cells as well as its ability to inhibit cell migration and its antiangiogenic capacity without toxic effects on HUVEC and HEK 293 normal cells. [38,39]. Other studies have also shown that the association of SOX17 (SRY-Box Transcription Factor 17) with BR2 sharply increases the expression levels of the *Klotho* tumor suppressor gene, also suppressing the migration potential of cancerous cells [39].

We show here that the combination of 2-AEH_2_P and the BR2 peptide is cytotoxic toward TNBC cells, inhibiting cell proliferation for exposures up to 72 h, therefore evidencing a long-lasting pharmacological effect of the combo. The 2-AEH_2_P and the BR2 peptide combination was also effective in inhibiting the proliferative and invasive properties of the tested tumor cells, but not normal cells. Studies developed by our group showed similar, albeit less effective, results for 2-AEH_2_P, reducing the proliferative and invasive capacity of B16-F10 melanoma cells and MCF-7 breast adenocarcinoma cells, and as discussed previously, similar effects are observed for the BR2 peptide when associated with other drugs, either potentiating the effect of allowing greater intracellular availability of the combined compounds [11,13,14,39,40].

Corroborating the change in the proliferative potential of tumor cells, there was a reduction in the number of cells expressing PCNA upon 2-AEH_2_P and the BR2 peptide treatments. PCNA is known for its role in DNA replication and repair, being identified as an interesting target for cancer therapy [41,42]. Recent data indicate that PCNA functions as a scaffold protein outside the nucleus, acting as an important regulator of vital mechanisms such as apoptosis, immune invasion of tumor cells, glycolysis, and cell signaling involving the PI3K/Akt/mTOR and MAPK pathways [43]. Interestingly, cell-penetrating peptides containing the PCNA-interacting motif APIM have been demonstrated to affect apoptosis, cell signaling, and DNA repair in mammalian cells and to possess anti-cancer efficacy in various preclinical cellular and animal models [43,44,45,46].

One of the main responses to drug-induced DNA damage is the increase in p53 expression that leads to the activation of the intrinsic apoptotic pathway [47,48]. The possible mechanism by which 2-AEH_2_P and the BR2 peptide-induced cell death in a human breast adenocarcinoma cell line involves the modulation of p53 protein activity, which was probably responsible for the increase in caspase 3 and later caspase 8 protein expression. It is worth mentioning that we have previously obtained results that corroborated these data, with a reduction in the expression of the Bcl-2 protein, a significant increase in Bax, and release of cytochrome C after exposure to 2-AEH_2_P and BR2 peptide [34]. In this study, treatment with 2-AEH_2_P did not promote an increase in p53 expression or of proteins that have transcriptional activation regulated by p53, like Bax and p21.

An important factor in tumorigenesis is angiogenesis, a phenomenon directly involved in tumor progression and metastasis, and the angiogenesis pathway has long been viewed as a relevant therapeutic target in cancer therapy. The vascular endothelial growth factor (VEGF) pathway is an important regulator of tumor angiogenesis [49]. Some studies have demonstrated that peptides mimicking the binding sites of VEGF significantly inhibit tumor development and progression to metastases in a 4T1 tumor cell model [49,50]. Interestingly, several studies have shown that after undergoing proteolytic cleavage, the RLLR terminal portion of the BR2 peptide binds to integrin-1 [51]. Neuropilin-1 is an angiogenic co-receptor of VEGF-A, implicated in the epithelial-mesenchymal transition (EMT), directly related to increased survival of tumor stem cells and formation of highly aggressive and vascularized tumors [52,53]. Han et al. have shown that blocking neuropilin-1 can inhibit cell proliferation and metastasis in addition to promoting apoptosis in this group of cells [54]. Thus, the reduction of VEGF after 2-AEH_2_P and the BR2 peptide treatments may be linked to inhibition of the VEGF/Nrp-1 pathway, consequently leading to a reduction in cell proliferation and migration [55].

## 5. Conclusions

We show here that the 2-AEH_2_P+BR2 combination was significantly more cytotoxic toward TNBC tumor cells compared to the compounds used alone, leading to reduced proliferative potential and cellular migration. The MDA-MB-231 human breast adenocarcinoma cell line exhibited the highest sensitivity to all treatments, with important morphological changes, especially in mitochondria, which lost their integrity with an increase in cells with inactive mitochondria or low mitochondrial electrical potential. Peptide BR2 and 2-AEH_2_P+BR2 were effective in activating regulated cell death in MDA-MB-231 tumor cells, possibly modulating the intrinsic pathway of cell death by increasing p53 expression. The pro-apoptotic property of the treatment was evidenced by the increase of cells expressing caspases 3 and 8 and the concomitant reduction of their mitochondrial electrical potential. A considerable decrease in the proliferative rate of tumor cells was observed with all treatments, validated by the reduction in the number of cells expressing the PCNA marker. 2-AEH_2_P+BR2 combination also induced a reduction in the number of cells expressing the VEGF receptor, which could be directly linked to the ability of the RLLR terminal portion of the BR2 peptide to bind integrin-1 and inhibit the formation of the VEGF/Nrp-1 complex. Combined, our preclinical data indicate that the 2-AEH_2_P+BR2 combination constitutes an innovative therapeutic strategy for breast cancer treatment, and clinical testing is warranted.

## Data Availability

The data presented in this study are available in this article.
